# Thinking about returning home from a protected haematology unit: confronting healthcare providers’ representations and patients’ experiences in a qualitative study

**DOI:** 10.3389/fpsyg.2025.1610567

**Published:** 2025-11-24

**Authors:** Sandrine Letrecher, Yasmine Chemrouk, Livia Sani

**Affiliations:** 1 Université de Caen Normandie, Caen, France; 2 CHU Orléans, Orléans, France; 3 Université de Strasbourg, Strasbourg, France

**Keywords:** healthcare providers, haematology, isolation, patient care, home

## Abstract

**Background:**

The psychological impact of protective isolation in haematology has been widely studied during hospitalization, but little is known about what happens when patients return home.

**Aim:**

Drawing on two qualitative studies conducted in French hospitals—one with patients and one with healthcare providers—this article explores how both groups experience the transition out of protective isolation. The focus is on the psychological challenges of returning home, shifts in identity and family roles, and the emotional dynamics between patients and staff.

**Methods:**

This article presents findings from two qualitative studies in clinical psychology. The first involved 10 non-directive interviews conducted with patients following their discharge from a Protected Haematology unit; the data was analyzed using grounded theory and narrative analysis. The second study comprised 12 semi-structured interviews with healthcare professionals, analyzed through interpretative phenomenological analysis (IPA).

**Results:**

Returning home poses significant challenges for patients and their families. Sterile constraints often persist, and hospital discharge does not necessarily signify recovery. At home, patients must navigate ongoing difficulties, including strict hygiene protocols, dependence on others, frequent medical appointments, and profound identity shifts related to illness. Healthcare professionals face a double bind: they are expected to celebrate the patient’s discharge while recognizing that it does not mark the end of isolation. In end-of-life contexts, they may emotionally distance themselves from patients—a defense mechanism that protects them from the grief associated with high mortality rates in oncology and haematology.

**Conclusion:**

The findings underscore the importance of dedicated spaces for reflection and dialogue—at home, to support patients and their families, and within hospital settings, to enable healthcare professionals to process potentially traumatic experiences.

## Introduction

Isolation into somatic care can occur in two situations. First, patients with a pathogenic infection must be isolated to prevent the spread of infection to others (septic isolation). Second, patients with severe immunodeficiency are isolated to prevent infection from the hospital environment, other patients, visitors, or their caregiver (protective isolation) (Nonnenmacher, 2019).

In oncology and haematology, the treatment pathway (chemotherapy, bone marrow transplant) can lead to several consecutive hospitalizations in the protected units. Most patients are admitted as emergency cases after consulting their primary care physician due to persistent fatigue or fever. They are often in a state of psychological shock, facing a dual burden (Proia-Lelouey and Letrecher, 2018): the diagnosis of a life-threatening cancer and the requirement for approximately 5 weeks of isolation (Chantepie, 2025).

Access to the sterile unit is not as easy as in a conventional hospital ward. Upon entering the changing room, patients are required to remove their clothing and take a final shower prior to isolation. If the hospital admission was scheduled, they are provided with sanitized clothing upon entering their room. Afterwards, a member of staff helps them sort out their personal effects and the food they have brought, separating what is permitted from what is prohibited. The unit has systems in place to control all possible sources of contamination, including air, water, and medical equipment. Each room has higher air pressure than the corridor and the airlock area, ensuring that when doors are opened, air flows outward rather than inward. The door must therefore always be kept closed. There are no toilet facilities in the rooms, but a commode and a washbasin are provided. To preserve their privacy, patients can use a folding screen. Each room is equipped with a television, a refrigerator, an exercise bike, and a Wi-Fi connection. For the entire duration of hospitalization, access to the room is limited to two people designated by the patient, who must depart by late afternoon. Children under the age of 15 years are not allowed in the unit (Letrecher and Proia-Lelouey, 2019).

Induction refers to the phase of initial treatment with intensive chemotherapy, leading to bone marrow aplasia. The aim here is to achieve remission, i.e., the disappearance of clinical and biological signs of the disease. During this period, the patient is highly fragile and vulnerable to various infections. Once remission has been achieved, however, abnormal leukemic cells persist in the bone marrow. Consolidation is the treatment phase designed to maintain remission by reducing the number of residual leukemia cells. It involves one or more courses in chemotherapy. Intensification through Hematopoietic Stem Cells (HSC) transplantation constitutes the third stage of treatment, typically proposed after the consolidation phase.

In the allograft clinic, graft-versus-host disease is common (Ferrara et al., 2009), with a reported incidence ranging from 20 to 50% (Madelaine and Faure, 2020). Both the internal and external surfaces of the patient’s body are affected. Externally, there are skin rashes, burns, redness, and massive scaling. Internally, excessive dryness affects the mucous membranes of the mouth and throat, causing ulcers, and similarly affects the mucosal tissues of the lungs and vagina. Eating becomes difficult and feeding tubes are frequently used. One-third of patients were found to be malnourished (Bassim et al., 2014).

Beyond these physical complications, patients also face significant psychological challenges. The psychological impact of haematological malignancies is well documented (Brett et al., 2023), and substantial research has been conducted on units specifically designed for adolescents (Ricadat et al., 2018).

Few studies have examined the combined aspect of oncology management and the effect of confinement in a sterile unit. Existing research mostly focuses on the accompaniment of children (Kellerman et al., 1976) and, more recently, adults (Barron et al., 2008).

Total isolation is detrimental to human psychological wellbeing (Gilmartin et al., 2013), as demonstrated by an extensive body of literature summarized in meta-analyses of qualitative studies (Vottero and Rittenmeyer, 2012; Biagioli et al., 2017). It is associated with higher rates of depression, anxiety, and anger. In onco-haematology, patients’ bodily integrity is threatened in two ways: by the loss of personal freedom and by the physical and psychological side effects of both the disease and its treatment (Letrecher, 2024). As Chemrouk et al. (2022) observe, the violence inflicted by illness and that caused by confinement mirror and reinforce each other.

Healthcare providers play a crucial role in addressing the psychological impact of isolation. Literature emphasises the critical need for tailored psychosocial and psychological care. One of the earliest forms of support includes mediated therapies, particularly art therapy. It is recommended for both children (Klein, 1955) and adults (Lane and Graham-Pole, 1994). Several authors highlight the non-verbal dimension, which, thanks to its metaphorical potential, offers therapeutic benefits to patients (Gabriel et al., 2001; Greece, 2003; Adcock, 2008; Forzoni et al., 2010; Agnese et al., 2012).

According to Janiaux (1992), in these medical contexts, patients often struggle to make sense of their experience. A psychotherapist is therefore needed to help them find someone to speak to and to provide a safe space in which their words can be understood and supported. Ibled (2011) highlights the major risks of subjective destitution and regression to archaic experiences of distress. These can lead patients to a melancholiform state dominated by the collapse of identity. She mentions the risk of over-investment by the care team, which may exacerbate difficulties following patient discharge. She describes the clinician’s role as that of a companion, enabling the patient to regain a breath of freedom. Finally, Ricadat et al. (2017) emphasize the need to support caregivers. For instance, adolescents’ opposition to care can be understood as an attempt to maintain their autonomy and preserve their subjectivity, which demands considerable patience on the part of caregivers.

McGrath (2015) advocates for psychosocial support during hospitalization. Patients are particularly vulnerable emotionally because of the shock stemming from their diagnosis and the confrontation with a potentially life-threatening condition. During this period, they may also be separated from their families, especially when these families live in rural areas and must travel to metropolitan centers to visit their hospitalized relative. Parkinson (2006) reports that significant distress can emerge at the time of discharge, as patients no longer have the strength to care for themselves and must rely on others for support. Dunn et al. (2016) argue for continued care post-hospitalization. They address the notion of a significant existential crisis, marked by a disruption in patients’ lives, the impacts of which persist well beyond the acute phase of the illness; especially in the early stages (Raphael et al., 2017).

Recent research explored the institutional connection between hospitals and homes by establishing hospital care platforms (Nipp et al., 2022) and emphasizing the need to provide support at home (Bennich et al., 2022). The nature of the provided support varies: it may be technical support for symptom assessment and management (Nipp et al., 2022), or educational around portable pump chemotherapy (Bennich et al., 2022), or psychosocial support, in which nurses intervene to strengthen the relationship between patients and their caregivers (Serçe and Günüsen, 2021). With its diverse approaches, this research offers promising avenues for improving patient care and wellbeing.

We aim to investigate the psychological experiences of transitioning from confinement, focusing on the perspectives of both patients and healthcare providers. Additionally, we seek to study the challenges faced upon returning home. As such, we plan to present and analyze two qualitative research studies in clinical psychology conducted in two French hospital settings.

The first qualitative study examines the psychological adjustments of patients hospitalized in protected haematology units upon their return home, following the emotional and physical impact of the initial diagnosis and hospitalization. Far from the ‘protective’ hospital environment and the support of healthcare staff, patients must reorganize their lives, redefine their roles, and reconnect with loved ones. Four hypotheses structure this research. The care modalities inherent to the haematology unit have psychological consequences for patients. Particularly, the nature of the relationships maintained with loved ones during hospitalization affects the experience in the unit. The announcement of discharge disrupts the temporary psychological adjustments made during hospitalization. A psychological support system based on transition theory may help sustain the patient in restoring their capacity for symbolization, which was previously undermined by the announcement of the illness and the care journey.

The second study focuses on the day-to-day care of patients with haematological malignancies in a protected sector. This environment, beyond being private, is subject to significant uncertainty. Patients in the intensive curative phase of treatment are at risk of death, both due to the aggressiveness of their pathology and to their undergoing treatments. This inductive exploratory study is purely qualitative. It aims to study how caregivers’ relationship with patients is shaped by uncertainty and hope.

## Methods

This section outlines the design and methodological features of two qualitative studies conducted in French Protected Haematology units. Both studies aimed to explore the psychological experiences and emotional dynamics associated with protective isolation and the transition back home, from the perspectives of patients and healthcare providers. Using distinct but complementary approaches—grounded theory, narrative analysis, and interpretative phenomenological analysis—the studies were embedded within a constructivist epistemology and adhered to SRQR reporting guidelines (O’Brien et al., 2014) (see Supplementary File 2).

### Study 1: psychological assistance in a protected haematology unit

Our study aims to test a system of psychological assistance in a Protected Haematology unit based on transitionality (Anzieu et al., 1979).

Ten patients, comprising five men and five women, aged 18–70 (mean age, 51.8), were interviewed and completed a questionnaire.

Participants were selected to represent a diverse range of ages, genders, and clinical conditions. Data collection continued until thematic saturation was achieved, meaning that no new themes emerged from the additional interviews.

The questionnaires enabled us to compare two modes of care (A and B) in terms of the psychological benefits they provided to the patients. We divided the patients randomly into two groups.

The recruitment process took place in the hematology department. Patients who expressed an interest in receiving psychological support—either spontaneously or following a suggestion from the care team—were referred to psychologists from the Mobile Palliative Care Unit. Recruitment continued until two cohorts of five participants each (Groups A and B) were formed.

During an initial bedside interview, the psychologists assessed eligibility criteria and explained the study protocol. Patients were provided with detailed written information and given 1 week to consider participation. Those who agreed were invited to a second meeting, during which any remaining questions were addressed, informed consent was obtained, and group allocation was disclosed. Patients who declined participation were nonetheless offered standard psychological care outside the research protocol.

*Group A*: A clinical unstructured interview was conducted weekly with 5 patients during hospitalization.

*Group B*: A clinical unstructured interview was conducted weekly with 5 patients during hospitalization, then on discharge, and every week at home for the following 2 months.

The questionnaires were co-constructed with the healthcare staff^1^. A nurse, the patient, and the research investigator each completed it anonymously at 3 time points: upon admission to the sheltered unit, at hospital discharge, and 2 months thereafter. One out of 10 patients passed away before filling out the third questionnaire, resulting in nine questionnaire responses.

Data were collected over a one-year period (May 2019–April 2020), concluding with the onset of the COVID-19 pandemic, and were triangulated through 125 clinical interviews conducted with patients in protected units and later at home. We excluded individuals with severely impaired cognitive functions, those who did not speak French, or those under legal guardianship. The researcher (Letrecher), a PhD and clinical psychologist with experience in haematological care, conducted, recorded, and transcribed all unstructured interviews. While this background facilitated trust and openness in clinical conversations, it also required careful attention to reflexivity. Reflexive memos and peer debriefings were used throughout to monitor emotional involvement and mitigate interpretative bias.

This study was conducted from a constructivist and interpretative epistemological perspective, which acknowledges the co-construction of meaning between participants and researchers. Our approach focused on how individuals give meaning to their lived experiences, particularly in the context of illness and recovery. Grounded theory and narrative analysis were selected to explore processes of identity transformation and symbolic reorganization, as expressed through discourse.

Grounded theory (Glaser and Strauss, 1967; Charmaz, 2008) is an internationally recognized method that is widely used (Charmaz and Mitchell, 2001). It is characterized by the conceptualization of empirical data (Méliani, 2013), and aims to develop the capacity to conceptualize new data (Chun Tie et al., 2019). Its inductive and reflexive aspects are particularly well suited to psychological practice (Olmos-Vega et al., 2022), and Charmaz (2008) used it to investigate the nature of psychological interventions in palliative care.

Since the 1980s (Mitchell, 1981), narrative analysis has played a criticalin the international scientific debate. Despite the diversity of approaches, many scholars acknowledge the influence of the French philosopher Paul Ricoeur. Ricoeur suggested that narrative identity is the “fragile offspring of history and fiction” (Ricoeur, 1985, p. 355), suggesting that the story of a life is constitutes a fusion of fiction and history (Ricoeur, 1988).

The National Committee approved this research for the Protection of Persons.

All interviews were recorded, transcribed verbatim, and anonymized. Personal identifiers were removed, and all data were stored securely in encrypted institutional servers in compliance with GDPR and ethical research standards.

We relied on the procedures of the Thematic Apperception Test (Shentoub, 1990) to analyze the discourse line by line and code it thematically.

To enhance trustworthiness, we used triangulation of data sources (patients and professionals), double coding by two independent researchers, and maintained an audit trail of analytic decisions. Reflexive memos were used to monitor the researchers’ influence on interpretation.

### Study 2: healthcare providers’ experience in a protected haematology unit

Our study aims to shed light on the dynamics that underlie the caregiver-patient relationship in moments of thoughtlessness and ambivalence. It is better to understand the defensive, adaptive, and relational strategies — both conscious and unconscious — employed by these care providers as they navigate the “extreme” situations inherent to their professional practice.

Regarding recruitment, an information notice was distributed to the entire care team. In practice, participants were primarily professionals with whom previous professional collaboration had taken place. No personal relationships were involved. All interviews were conducted in a neutral setting, outside the clinical ward.

Twelve female care providers working in a Protected Haematology unit of a general hospital participated in the study. The sample consisted of six nurses (mean age, 31.2 years; mean length of service, 8 years) and six orderlies (mean age, 42.5 years; mean length of service, 5 years). There were no male caregivers in this unit, which explains the lack of gender diversity (see Limitations).

Participants were selected to represent a diverse range of roles and seniority levels. Data collection continued until thematic saturation was achieved, when no new themes emerged from the final interviews.

Data were collected over a one-year period (May 2019–April 2020), ending with the onset of the COVID-19 pandemic.

Twelve semi-structured interviews were conducted outside the care setting, using a grounded theory guide developed from preliminary research in the same department. After obtaining their informed consent, all interviews were conducted, recorded, and transcribed (YC).^2^ Interpretative phenomenological analysis (IPA) seemed the most appropriate method for studying the subjective experience of caregivers faced with different types of uncertainty. It was used with NVivo software. Developed by Smith in 1996, interpretative phenomenological analysis (IPA) has become a primary qualitative method in psychology, particularly in health and clinical psychology.

It involves the in-depth study of an individual’s lived experience, based on their own account and personal understanding of that experience. IPA is based on and via the person’s subjective report of their experience. It draws upon the universal human tendency to self-reflection (Chapman and Smith, 2002; Smith et al., 1997) and the interpretation of one’s own experiences in a way that makes sense to them. Thus, within this framework, subjective data are no longer considered as biased, but rather as the narrative material the method will solicit and analyze. IPA through the analysis of these narrative enables us to understand the meanings individuals attribute to their experience and the processes used to attribute this meaning to a specific experience.

This research focused on the prognostic uncertainty inherent to the specificity of this type of haematological disease and its impact on the caregiver-patient relationship.

The University of Strasbourg approved this research.

All interviews were recorded, transcribed verbatim, and anonymized. Personal identifiers were removed, and all data were stored securely in encrypted institutional servers in compliance with GDPR and ethical research standards.

The researcher Chemrouk (2021), is a PhD and clinical psychologist with experience in haematology units. Her familiarity with the hospital environment may have facilitated rapport with participants but also required active reflexivity throughout the study. Field notes and regular peer discussions were used to identify and mitigate potential biases related to professional positioning and emotional involvement.

To minimize potential bias related to the researcher’s dual role, the analyses were reviewed by an experienced researcher.

### Objective of contextualization

During the first research project (Letrecher, 2021), all the nursing questionnaires, which were conducted 2 months after discharge, remained unanswered. The nursing staff explained that patients could not respond accurately due to the distance that had developed since their discharge. However, these questionnaires were designed with partial input from service professionals. Hence, it is essential to compare our data with those from a study conducted by a colleague in another French hospital (Letrecher, 2021), which focuses on the experiences of healthcare providers in the haematology sector. This comparison could shed light on this healthcare provider’s inability to complete the section of the questionnaires related to the at-home experience.

We intend to rely on the patient discharge questionnaires (Research 1) and compare the verbatim responses of patients (Research 1) with those of healthcare providers (Research 2).

## Results from study 1: patients’ experiences of returning home following hospitalization in a protected haematology unit

Questionnaire results are presented in tabular form^3^. Verbatims excerpts were included to humanize the results and highlight the expressive power of the participants’ words. The verbatims have been translated into English by the authors.

Two months after leaving the protected area, patients’ responses to the questionnaires raised questions about the relevance of the term “leaving” to describe the patients’ situation: indeed, half of them had returned in conventional units (4/9),^4^ while four were only at home-but only for 2 weeks and only one patient had been at home for 2 months, and she was the only one without a cancerous condition (aplastic anemia). Their verbatim testimonies reflect this ambiguity: “It feels like we. We leave, and then. We dive back in” (Patient No.1). It is as if words no longer have meaning: “So, leaving for good, what does that mean. I wondered.” (Patient No.2).

Nevertheless, returning home, even briefly, was positively evaluated by the patients (8/9). Thus, Patient No.3 appreciated regaining a certain level of privacy at home, echoing the importance of personal care, such as “getting a shower,” “going to the toilet,” and “shaving.”

However, home complaints are diverse and reveal a sense of persisting hospital constraints. Patient No. 4 felt like he was “stepping back into a trap,” namely, his upstairs bedroom. He felt like “a pariah.” “As if I were contagious, when it’s the opposite, others can infect me.”

A third of the patients complained about the absence of healthcare personnel (3/9) and felt somewhat lost at home despite the presence of some of their family members (2/3). Patient No.5, a single man living alone at home, remembered how, during his hospitalization, “the nurses held my hand all night when I had a fever. and now nothing.”

The interviews highlight the importance of the support provided by home care nurses. Patient No.1 emphasized the reassurance of having a nurse living nearby, “just next door.” Other patients insisted on feeling constrained by the schedules of the home care professionals. “Well, I have to be available to them” (Patient No.3). Overall, the home, a place of intimacy, was experienced by the patients as invaded by illness (7/9), to varying degrees. For Patient No.5, this was intolerable- He described his home as a “sanctuary” which was disturbed by the nurse’s weekly “No, but in my home, it’s my home. I do not like people meddling in my affairs.”

Most patients reported a change in their relationship with their loved ones (8/9). Two patients perceived this positively; these were individuals they did not interact with daily. Patient No.1 mentioned that he has rekindled a relationship with a sister-in-law from whom he had been estranged for several years. She said to me ‘A serious illness makes you think.’ Six patients negatively evaluated the change in their relationships with loved ones they interacted with daily, such as their spouse and children.

Patient No.7 described a domestic apocalyptic scene: “The washing machine broke down,” “there was water everywhere.” He was then a “spectator,” “tied” to his infusion stand, feeling like “good for nothing,” while his wife and younger son “struggled with the flood.” Support from adult children displaces patients from their active role.

### Three themes emerged from patient interviews

Each interview was transcribed and coded according to the themes discussed. Only the themes related to our topic and present in most patients are included.

#### Home becomes a place of traumatic reminiscences linked to their hospitalization

The idea of returning to the unit for further treatment is unbearable for most patients: “And then it’s going back to the sterile room!” (Patient No.2). Patient No.1 could not stand his wife’s ringtone: “That ringtone. it affects me.” which reminded him of painful separation moments when she set her phone alarm for her parking ticket. These moments were terrible, and he asked his wife to change the ringtone.

#### The unit is also a place where privacy is lost and exposure to daily shame is inevitable

Upon returning home, Patient No.8 spoke of what he called “humiliations”: “In the unit, you have to separate your urine; they count it.”; “I do not know if you know what it’s like.”;"Honestly, it’s tough, really, it’s tough.”

#### Irreparable loss

Finally, because home is a marker of an individual’s identity, it’s also a place to realize irreparable loss. Nine months into his treatment, Patient No.4 talked about how “he cannot stand his own image”; “(he) does not recognize (himself).” He reported that at home, before taking a shower, he turns on the hot water to the maximum, leaves the bathroom, and waits until there the mirror is heavily steamed.

## Results from study 2: healthcare providers’ perspectives on patient discharge and transition to home

Care providers’ remarks revealed a similar difficulty in using the term “leaving the ward”: “Sometimes we discharge them, and not even 2 or 3 days later, they come back due to an emergency” (Caregiver No.1). It becomes a metaphor that questions the possibility of recovery: “You do not know if they will get better or not, if they will leave the ward or not; or if they will simply get through it!” (Caregiver No.2).

They witnessed their patients’ apprehension about returning to autonomy: “afraid of not knowing how to behave at home” (Caregiver No.1). They reported they must accompany this discharge by maintaining contact through phone calls: “You can call us. We are here to answer you. We know it’s not easy at home” (Caregiver No.3).

They imagined the physical distance from loved ones by identification: “You will go home, you will have to disinfect everything,” and “should not be in contact with family members who may be ill or young children” (Caregiver No.3).

These care providers were also aware of the risks of excessive protection their loved ones that come with returning home. “Sometimes, there are those who go to extremes, even to the point of not wanting to sleep with their partner.” (Caregiver No.1).

The fear of not doing enough can lead to drastic measures, such as “repainting the entire house” to accommodate a patient (Caregiver No.3). They recognize how difficult it is: “It’s not easy to support someone who is undergoing treatment, especially with all the complications at home, cleaning, shopping, and food.” (Caregiver No.4).

In the years following the end of treatments, the disease is omnipresent to the point where they question the relevance of the term “life”: “We tell them, you can go on a weekend, but you need a nearby hospital, take your blood group card, your medical records, your prescriptions. It’s not a life; they never break free from the illness” (Caregiver No.3).

Caring for and identifying with patients involves a struggle against their de-subjectivation, which threatens them: “When I notice that, sometimes I dig a little into their private lives. But it’s not out of curiosity; rather it’s intended to give the person in front of me a bit of their identity back. I ask: in life, what do you do, what do you like?” (Caregiver No.4).

Deep emotional bonds form between care providers and patients due to the intensity and duration of care in the ward, which Caregiver No.5 acknowledged indirectly. “He says he has special relationships with some, it warms the heart even if we do not take the place of family.”

These bonds are even more potent when patients are isolated from their loved ones: “These are patients we want to take care of even more because they are alone, we had a rather buddy-buddy relationship. We teased each other, we laughed” (Caregiver No.5).

Returning home is also discussed when it occurs without the prospect of a cure. Caregiver No.5 identified with patients in the context of the announcement of treatment cessation: “It’s like they are telling you, we are trying to put ourselves in the person’s shoes, you have this, it does not heal, but it’s very bad prognosis, but we will not do anything for you, go home.”

Their discourse then reflects an idealization of home as a place where the patient can enjoy “the time left”: “I remember patients for whom I said they would have been better off going home, enjoying it because they ended up in a badly (Caregiver No.6). The need to maintain hope among professionals is evident, as they appear to be in denial of the end-of-life context: “This little time left; I would like them to enjoy it. Mr. L. when he was discharged. I told him: enjoy it, if there are things you wanted to do, do them now” (Caregiver No.5).

The announcement of death generated ambivalence among the care providers. They expressed regret for not accompanying the end of life due to the intensity of the bonds formed while, simultaneously recognizing that this separation offered them protection and reinforced their professional status: “It’s good because it also protects us. We do not do end-of-life care. But alongside that, it’s frustrating” (Caregiver No.7).

Not accompanying the final moments allows them to maintain a denial of death. Caregiver No.2 remembers an old patient: “Her husband showed me a picture of the grave, and he started crying. Oh, it. It tore my heart apart because I’m not here to, you know, it made me sad, what do you want to say? But he needed it. Sometimes, I prefer not to hear from loved ones because it makes me sadder. I’m sad for them. For those who are left, it’s complicated.”

## Discussion

We propose to begin with a summary of the study results.

### Study 1

The distinction between “home” and “hospital” fails to reflect patients’ real experiences, as the care pathway is often unstable. Illness and hospitalization disrupt family dynamics, and care routines hinder intimacy. The death of others is a recurring theme in patients’ stories, through which they reaffirm being alive. Transitional follow-up with non-directive interviews helps prevent passivity, allowing patients to reclaim meaning in experiences beyond their control. This approach also supports relatives at home.

### Study 2

Protective isolation is central to ethical tensions, particularly when caregivers idealize the concept of home. Caregivers often grapple with the deprivation experienced by patients, especially when their condition worsens and their prognosis becomes uncertain. This uncertainty regarding survival can challenge caregivers’ sense of purpose. In the palliative phase, when caregivers no longer hold out hope for the patient’s recovery, the notion of home transforms. It becomes idealized and is often described in caregivers’ discourse as the heart of the family—a place of life and even joy. As a result, hospitalization becomes a place of deprivation rather than a source of hope for recovery.

It is a matter of “bearing” constraints in the ward for both patients and healthcare providers. This verb reflects their shared experience of an *extreme situation* (Bettelheim, 1960). During hospitalization, the intimacy between patients and staff takes on a maternal quality (Winnicott, 1956). Caregiver 5 describes how she “nurtures” certain patients, echoing the emotional closeness that is often required in such contexts. This protective cocoon replaces the psychological and physical layers shattered by illness and care (da Rocha Rodrigues and Séchaud, 2019).

Separation from professionals at discharge is experienced as a rupture. The regression induced by hospitalization (Letrecher and Proia-Lelouey, 2019) leaves patients ill-prepared for autonomy. Even at home, they remain dependent and bound to strict protocols. Our findings reinforce earlier research highlighting the psychological vulnerability following discharge (Dunn et al., 2016; Raphael et al., 2017). In Patient No. 4’s case, the violence of the psychic upheaval ultimately led to psychiatric hospitalization.

Care providers are aware of these difficulties but face a double bind (Bateson et al., 1956): they must celebrate discharge as a success while warning patients of ongoing risks. As Caregiver No. 5 encourages patients to “enjoy life,” Caregiver 3 reminds them not to get too close to their loved ones. This paradox generates emotional strain, as also described by Bodschwinna et al. (2022), and may contribute to their reluctance to complete post-discharge evaluations (research one) or to the high turnover that disrupts continuity of care.

To cope with emotional overload, professionals often develop psychological and institutional distance. This is especially evident in end-of-life contexts, where some caregivers idealize home as a space of peace and autonomy—“Do the things you wanted to do now,” says Caregiver No.5. Yet such messages may obscure the reality of impending death. As Proia-Lelouey and Boissel (2023) note, this rhetoric of “hopeful autonomy” can mask a more profound need for emotional processing within care teams.

At the same time, care relationships can provoke powerful countertransference reactions. Caregiver No.5 admits she “cannot stand some patients anymore.” This unspoken reality reveals how helplessness, despair, and exposure to life-threatening illness can clash with the caregiver’s ideal (Winnicott, 1949; Ciccone, 2014; Kahr, 2020). Professional identity is tested, and distance becomes both a defense and a burden.

The institutional separation between hospital and home helps caregivers manage grief. Yet it comes at a psychological cost, as it suppresses the ongoing impact of care. Caregiver No.2”s pain upon hearing from the family of a deceased patient—"it tore my heart apart”—reminds us that emotional residue persists well beyond the clinical encounter. In metropolitan France, the five-year survival rate for acute myeloid leukemia remains just 27% (Coureau et al., 2021), and professionals are exposed to repeated loss. This long-term exposure should be recognized as a potential source of post-traumatic stress (Letrecher, 2024; Kahr, 2020).

From the patient’s perspective, the home is ambivalent: a space of intimacy and intrusion. Our findings echo those of Serçe and Günüsen (2021), who describe how home care can either reinforce or strain familial ties. For Patient No. 6, the nurse’s presence disrupted his sense of sanctuary; for Patient No.1, it was reassuring. This tension illustrates the ambivalence of home as both hospitable and hostile (Derrida, 1999; Lassagne et al., 2023).

In this context, narrativity becomes essential. Their stories express the conflict between past identity and present fragility. Since the 1980s (Mitchell, 1981), narrative analysis has been a significant contributor to the international scientific debate, particularly in understanding the construction of identity through storytelling. Ricoeur’s concept of narrative identity (1985, 1988) frames the patient’s attempt to reconstruct coherence amid disruption, as when Patient No.4 fogs the mirror before showering, refusing to confront a self he no longer recognizes.

Likewise, healthcare providers create their narratives to manage emotional ambivalence and loss. Idealizing home as a place of redemption or rest is one way to maintain hope in the face of terminality. But as Caregiver No.2’s account shows, death still echoes—quietly, painfully—well beyond the ward.

### Limitations

This study has several limitations. First, the relatively small sample size and the focus on two specific French hospitals may limit the transferability of the findings to other settings and populations. Future research should include broader geographic and institutional contexts to enhance generalizability. Second, the healthcare professional interviews involved only female participants from a single department, potentially limiting the representation of gendered and professional perspectives; future studies should aim to include male professionals and other roles. Third, although we attempted to mitigate interviewer–analyst bias through double coding, the dual role of the researcher may still have influenced data interpretation. Developing external coding teams or triangulation with participant validation could further strengthen methodological rigor. Finally, the absence of a longer-term follow-up restricts insight into how psychological and relational processes evolve; future longitudinal designs (e.g., 6–12 months post-discharge) could provide a clearer understanding of the trajectory of adaptation and identity reconstruction.

## Conclusion

In haematology, if entering isolation requires the patient to adapt—albeit in a less intuitive way—the same applies to exiting isolation and returning home. The treatment process is still ongoing, and significant constraints remain at home.

Professionals often conceptualize care systems and their coordination. However, their defense mechanisms do not always allow them to consider patients’ needs after discharge fully. The institution sometimes reinforces this divide. Care trajectories remain overly compartmentalized, particularly between the hospital and the home.

In Protected Hematology units, as in oncology more broadly, treatments are advancing and offering patients longer life expectancy. However, the psychological resources provided by institutions are not always sufficient to address clinical situations that resemble long-term palliative care.

In France, mobile palliative care teams—composed of a doctor, nurse, and psychologist—based in university hospitals do not always visit patients at home, even though such visits could help ensure continuity of care for those discharged from the hematology unit. During this transition, appointing a dedicated contact person within the hematology team to support patients could help maintain continuity and reduce the sense of disruption frequently reported by patients. The recent creation of the advanced practice nurse role in France could also help meet these needs.

And what about professionals? Psychology has long been interested in group therapy programs dedicated to them. Balint groups or structured supervision sessions offer professionals a space to manage their emotional overload and maintain their ability to provide care until the end of life. These groups offer the opportunity to express the silent burden of end-of-life care. Unfortunately, due to a lack of institutional will, no such programs existed in the institutions where we conducted our research.

From an ethical standpoint, it is essential not to separate patient health from staff wellbeing. Otherwise, scientific advances in oncology would amount to prolonging life at any cost.

## Data Availability

The original contributions presented in the study are included in the article/Supplementary material, further inquiries can be directed to the corresponding author.

## Footnotes

^1^See the summary tables in the appendix (QUESTIONNAIRES Tables A1–A6)

^2^Researcher’s initials.

^3^See the summary tables in the appendix (TOPICS ADDRESSED BY PATIENTS: Tables A1–A5).

^4^Four patients out of a total of nine patients.
