# Supplementary material for: Thinking about returning home from a protected haematology unit: confronting healthcare providers’ representations and patients’ experiences in a qualitative study
Source: Front Psychol. 2025 Nov 24;16:1610567. doi: 10.3389/fpsyg.2025.1610567 (PMC12683332; doi:10.3389/fpsyg.2025.1610567)
Supplement: Supplementary file 1 [file Supplementary_file_1.docx]

**APPENDICES**

**TOPICS ADDRESSED BY PATIENTS**

Table 1. **Representation of returning home**

| Representation of Returning home | Number of participants |
| --- | --- |
| positively evaluated by the patients | 8/9 |
| They appreciate returning to their daily routines | 7/9 |
| a sense of freedom | 7/9 |
| quality food | 6/9 |
| reconnecting with their family | 5/9 |
| reconnecting with their children | 4/9 |
| reconnecting with their spouse | 2/9 |

Table 2. **Types of constraints mentioned by patients as specific to the protected unit**

| Constraints specific to the unit | Number of participants |
| --- | --- |
| difficulty in tolerating confinement | 8/9 |
| suffering from restricted visits | 8/9 |
| alienation to the hospital routine | 5/9 |
| lack of quality in food | 5/9 |

Table 3. **Complaints at home**

| Complaints at home | Number of participants |
| --- | --- |
| sterile constraints | 3/9 |
| treatment side effects | 3/9 |
| prohibitions on daily activities | 3/9 |
| restrictions on certain leisure activities | 3/9 |
| dependence on family members | 3/9 |
| frequent returns to the hospital | 3/9 |
| persistent fatigue | 3/9 |
| inability to drive anymore | 2/9 |
| absence of healthcare personnel | 3/9 |
| feel somewhat lost at home despite the presence of some family members | 2/9 |
| miss the reassuring presence of healthcare professionals | 3/9 |
| the ability to ask them questions | 3/9 |
| benefit from their advice to adjust treatments | 3/9 |
| to benefit from their "human dimension | 2/9 |
| the home, a place of intimacy, as invaded by illness | 7/9 |

Table 4. **Change in their relationships with their loved ones**

| Change in their relationships with their loved ones | Number of participants |
| --- | --- |
| with their family in general | 5/9 |
| with their children | 4/9 |
| with their friends | 4/9 |
| with their spouse | 3/9 |
| with a colleague | 1/9 |
| negatively evaluate the change in their relationships with loved ones | 6/9 |

Table 5. **Psychological support**

| Psychological support | Number of participants |
| --- | --- |
| Psychological support has been found helpful | 8/9 |
| Psychological support has been found unhelpful | 1/9 |
| it allowed them to discuss the illness and treatments | 9/9 |
| and other concerns | 9/9 |
| felt understood | 9/9 |
| felt calmed | 8/9 |
| felt more centred | 7/9 |

**QUESTIONNAIRES**

Table 1. **Questionnaire themes**

| Representation of returning home |
| --- |
| Types of constraints mentioned by patients as specific to the protected unit |
| Complaints at home |
| Change in their relationships with their loved ones. |
| Psychological support. |

Table 2. **Representation of returning home**

| **Theme** | **Number of Patients** |
| --- | --- |
| Positively evaluated by the patients | 8/9 |
| Appreciate returning to daily routines | 7/9 |
| Sense of freedom | 7/9 |
| Quality food | 6/9 |
| Reconnecting with family | 5/9 |
| Reconnecting with children | 4/9 |
| Reconnecting with spouse | 2/9 |

Table 3. **Types of constraints mentioned by patients as specific to the protected unit**

| **Theme** | **Number of Patients** |
| --- | --- |
| Difficulty in tolerating confinement | 8/9 |
| Suffering from restricted visits | 8/9 |
| Alienation to hospital routine | 5/9 |
| Lack of quality food | 5/9 |

Table 4. **Complaints at home**

| **Theme** | **Number of Patients** |
| --- | --- |
| Sterile constraints | 3/9 |
| Treatment side effects | 3/9 |
| Prohibitions on daily activities | 3/9 |
| Restrictions on certain leisure activities | 3/9 |
| Dependence on family members | 3/9 |
| Frequent returns to hospital | 3/9 |
| Persistent fatigue | 3/9 |
| Inability to drive | 2/9 |
| Absence of healthcare personnel | 3/9 |
| Feeling lost at home despite family presence | 2/9 |
| Missing the reassuring presence of healthcare professionals | 3/9 |
| Ability to ask questions | 3/9 |
| Benefit from advice to adjust treatments | 3/9 |
| Benefit from their "human dimension" | 2/9 |
| Home invaded by illness | 7/9 |

Table 5. **Change in their relationships with their loved ones**

| **Theme** | **Number of Patients** |
| --- | --- |
| With family in general | 5/9 |
| With children | 4/9 |
| With friends | 4/9 |
| With spouse | 3/9 |
| With a colleague | 1/9 |
| Negatively evaluate the change in relationships with loved ones | 6/9 |

Table 6. **Psychological support**

| **Theme** | **Number of Patients** |
| --- | --- |
| Psychological support found helpful | 8/9 |
| Psychological support found unhelpful | 1/9 |
| Allowed to discuss illness and treatments | 9/9 |
| Allowed to discuss other concerns | 9/9 |
| Felt understood | 9/9 |
| Felt calmed | 8/9 |
| Felt more centered | 7/9 |
